# Hemophilia A: an ideal disease to correct *in utero*

**DOI:** 10.3389/fphar.2014.00276

**Published:** 2014-12-11

**Authors:** Christopher D. Porada, Christopher Rodman, Glicerio Ignacio, Anthony Atala, Graça Almeida-Porada

**Affiliations:** Regenerative Medicine, Wake Forest Institute for Regenerative MedicineWinston-Salem, NC, USA

**Keywords:** hemophilia, *in utero* transplantation, *in utero* gene therapy, fetal intervention, immune tolerance, mesenchymal stromal cells, sheep model

## Abstract

Hemophilia A (HA) is the most frequent inheritable defect of the coagulation proteins. The current standard of care for patients with HA is prophylactic factor infusion, which is comprised of regular (2–3 times per week) intravenous infusions of recombinant or plasma-derived FVIII to maintain hemostasis. While this treatment has greatly increased the quality of life and lengthened the life expectancy for many HA patients, its high cost, the need for lifelong infusions, and the fact that it is unavailable to roughly 75% of the world's HA patients make this type of treatment far from ideal. In addition, this lifesaving therapy suffers from a high risk of treatment failure due to immune response to the infused FVIII. There is thus a need for novel treatments, such as those using stem cells and/or gene therapy, which have the potential to mediate long-term correction or permanent cure following a single intervention. In the present review, we discuss the clinical feasibility and unique advantages that an *in utero* approach to treating HA could offer, placing special emphasis on a new sheep model of HA we have developed and on the use of mesenchymal stromal cells (MSC) as cellular vehicles for delivering the FVIII gene.

## Hemophilia A and the need for better treatments

Hemophilia A (HA) is the most commonly occurring inheritable deficiency of coagulation (Mannucci and Tuddenham, 2001). While the clinical severity of HA (based on FVIII plasma levels) can vary, up to 70% of patients with HA present with a severe, life-threatening phenotype, due to having less than 1% of the normal plasma levels of FVIII activity (Kay and High, 1999; High, 2003; Agaliotis et al., 2006). These patients suffer frequent spontaneous hemorrhaging, which leads to hematomas, chronic painful and debilitating arthropathies, and potentially life-threatening internal bleeding (Agaliotis et al., 2006). The current standard of care for HA is prophylactic factor infusion, which is comprised of regular (2–3 times per week) intravenous infusions of recombinant or plasma-derived FVIII to maintain hemostasis. While the availability of this protein-based treatment has greatly improved the quality of life and extended the life expectancy for many patients with HA, it is far from and ideal therapy. Patients are sentenced to a lifetime of multiple intravenous infusions each week, and are financially strapped with treatment costs that can exceed $300,000/year. Even among the ~25% of HA patients worldwide who are fortunate enough to have access to FVIII prophylaxis, approximately 30% will mount an immune response to the infused FVIII, forming inhibitory antibodies (inhibitors) to FVIII (Kaveri et al., 2007). In the best case scenario, these inhibitors simply reduce the effectiveness of subsequent infusions of FVIII; in the worst case scenario, they can lead to treatment failure, precluding restoration of hemostasis and putting the patient at risk of a life-threatening bleed. These significant shortcomings highlight the urgent unmet need for novel therapies that could promise longer-lasting correction, or permanent cure, of HA.

In contrast to current protein-based therapeutics, a single gene therapy treatment could promise lifelong improvement or permanent cure of HA; indeed, several aspects of HA make it an ideal target disease for correction by gene therapy (Lipshutz et al., 1999; Arruda, 2006; Ponder, 2006; Doering et al., 2007, 2009; Ide et al., 2007; Shi et al., 2008; Nichols et al., 2009; Tellez et al., 2010; High, 2011). First, FVIII, unlike the proteins that are missing/defective in many other genetic diseases, does not need to be expressed in either a specific tissue or cell type to produce a therapeutic effect. Although the majority of FVIII produced within the body is thought to be synthesized within the liver (Fahs et al., 2014), as long as FVIII is produced by cells that are close enough to the vasculature to secrete the synthesized FVIII into the circulation, FVIII can exert its appropriate clotting activity. Second, even if FVIII levels could be restored to only 3–5% of normal, this seemingly minimal change would be predicted to exert a marked clinical improvement and greatly improve the quality of life of patients with severe HA, since it would convert these patients to a moderate/mild phenotype. Conversely, even FVIII levels as high as 150% of normal should be safe. As such, FVIII has a very wide therapeutic window (Kay and High, 1999). Armed with this knowledge, the hemophilias were among the most promising, “Target 10,” group of diseases in the roadmap the American Society of Gene and Cell Therapy (www.ASGCT.org) recently provided to NIH director, Dr. Francis Collins.

## Sheep as a preclinical model of hemophilia A

A number of animal models have been developed to evaluate new methods of treating coagulation disorders, and also for preventing and devising way to overcome inhibitor formation. Fortunately, colonies of HA dogs in which spontaneous mutations occurred within the FVIII gene (Hough et al., 2002; Lozier et al., 2002) and FVIII-deficient mice produced via gene targeting/knockout (Bi et al., 1995) are both available to study the biology of FVIII and to begin developing/exploring gene-based strategies for treating HA. Pronounced therapeutic benefit has been demonstrated in multiple studies in the murine models (Gallo-Penn et al., 1999; Garcia-Martin et al., 2002; Reddy et al., 2002; Moayeri et al., 2004, 2005; Sarkar et al., 2004; Doering et al., 2007; Ide et al., 2007, 2010). Phenotypic correction has also been achieved in dogs with HA, but correction in this more clinically predictive model has proven to be much more difficult than in mice (Gallo-Penn et al., 2001; Scallan et al., 2003). However, despite the promising results that have been obtained in both these models, no clinical benefit has yet been seen in any of the clinical gene therapy trials that have been conducted to-date in human patients with HA. This is in striking contrast to the recent successes that have been reported in clinical gene therapy trials treating patients with hemophilia B (HB) (Nathwani et al., 2011); the reasons for the marked difference in the ability of gene therapy to correct HA vs. HB are not, at present, clear. Nevertheless, as a result of the disappointing outcomes thus far, no active clinical trials are currently ongoing in which gene therapy is being used to treat HA. This is especially vexing when one considers that roughly 80% of all hemophilia cases are HA.

The difficulties seen thus far translating success in animal models into therapeutic benefit in human patients highlight the importance of preclinical animal models that both precisely mimic the disease process of HA, and closely parallel normal human immunology and physiology. To this end, we used a variety of reproductive technologies to successfully re-establish a line of sheep (Bormann et al., 2006, 2007; Almeida-Porada et al., 2007; Sanada et al., 2008; Porada et al., 2010), originally described by investigators at the Swiss Federal Institute of Technology (Neuenschwander et al., 1992; Backfisch et al., 1994; Neuenschwander and Pliska, 1994), that possess a spontaneous mutation causing severe HA, which, if not treated immediately at birth, is fatal within the first hours/days of life. Upon re-establishing this line, we fully characterized the clinical parameters of this new model (Bormann et al., 2006, 2007; Almeida-Porada et al., 2007; Sanada et al., 2008; Porada et al., 2010). All 10 affected animals born thus far have presented with prolonged umbilical cord bleeding, protracted nail (hoof) cuticle bleeding time, bleeding following routine tail docking, and they have all experienced multiple spontaneous episodes of severe bleeding, including muscle hematomas, hematuria, and hemarthroses, all of which have promptly responded to infusion of human FVIII. Since aPTT can be fairly inaccurate when FVIII levels are very low, we had our collaborators at the BloodCenter of Wisconsin and at Emory University independently run a highly sensitive chromogenic assay to accurately quantitate the level of FVIII activity present in the circulation of these animals. The results of these assays quickly explained the severe, life-threatening phenotype we observed in this line of sheep, as FVIII activity was undetectable. Just like human patients with severe HA, these sheep experience frequent spontaneous bleeds into their “knees,” which, over time, produce crippling arthropathies that ultimately lead to decreased movement, difficulties walking, and eventually symptoms of pain even just to stand up. These recurring spontaneous joint bleeds make this line of sheep unique among animal models of HA. Also in similarity to human patients, some of these sheep developed inhibitors following administration of FVIII. However, since we were restricted to treatment with human FVIII (we had not yet cloned and sequenced ovine FVIII), it is not yet clear whether these animals will also make inhibitors to the ovine protein. An ongoing collaboration with investigators at Emory University has recently resulted in the successful cloning and large scale production of recombinant B domain-deleted ovine FVIII (Zakas et al., 2012), making it possible to address this important question and to construct gene therapy vectors encoding the native ovine sequence for testing in this valuable model.

In addition to studying the clinical picture of these animals, we also sequenced the entire coding region of the ovine FVIII gene to define the precise molecular basis for their disease. This knowledge of the nature of the disease-causing mutation enabled us to then design a PCR-based RFLP that allows us to unequivocally identify affected animals at birth and even *in utero*, using amniotic fluid-derived cells (Bormann et al., 2006, 2007; Almeida-Porada et al., 2007; Sanada et al., 2008; Porada et al., 2010). These studies revealed that HA in this line of sheep is caused by a frame shift mutation that introduces a premature stop codon part way through the FVIII coding region. Importantly, this type of mutation has also been reported in many human HA patients (Park et al., 2004). Since this line of sheep is, to our knowledge, the only animal model of HA yet described that possesses this type of mutation, these sheep provide a unique system in which to study therapies in this context.

While another large animal model of HA would already be of value, and the nature of the mutation present in these sheep makes them unique as an HA model, sheep possess many characteristics that make them an ideal preclinical model for gene therapy, especially in the context of HA. Firstly, sheep share many important physiological and developmental characteristics with humans. As a result, they have been used extensively in the study of mammalian fetal physiology, and the results obtained with this model have been directly applicable to the understanding of human fetal growth and development (Jeanblanc et al., 2014). In contrast to dogs, pigs, and many other large animals which tend to have large litters of offspring, sheep, like humans, typically give birth to only one or two offspring in each pregnancy. Secondly, sheep are similar in size/weight to humans, both at birth and as adults, making it possible to develop and test clinically relevant doses of vector/cells directly in this model prior to translating to the clinical arena. Thirdly, the development of the immune system during fetal ontogeny has been thoroughly delineated in sheep (Silverstein et al., 1966; Sawyer et al., 1978; Osburn, 1981; Tuboly et al., 1984; Maddox et al., 1987a,b,c), making this model ideal for investigating the immune facets of treating HA via gene therapy. An additional unique advantage to using sheep to study HA treatment is that in sheep, like human, the majority of the FVIII carrier protein, vWF, is stored/located within their platelets. This is in contrast to dog, in which vWF circulates free in plasma (McCarroll et al., 1988; Parker et al., 1991). This key difference makes the sheep the most clinically relevant large animal model in which to test the efficacy of platelet-targeted gene therapy approaches for treating HA (Shi et al., 2006, 2008; Shi and Montgomery, 2010; Montgomery and Shi, 2012). For these collective reasons, we feel that sheep are an especially fitting model in which to develop and test gene therapy treatments for HA.

## Feasibility and justification for treating HA prior to birth

Even if FVIII costs were reduced to the point that most HA patients could afford prophylaxis, these patients would still require recurrent, intravenous infusions throughout their lives, and still have a significant risk of treatment failure due to inhibitor induction. These problems, as well as many of the obstacles that have precluded gene therapy from curing patients with HA (and many other diseases) to-date, could likely be overcome/eliminated by performing gene therapy prior to birth. At the present time, HA can be diagnosed relatively early in gestation (10–12 weeks), just like many other genetic diseases. The ability to diagnose HA early in development makes it feasible to begin devising methods to try to correct this disease prior to birth. Fetal transfusions and *in utero* stem cell-based therapies have safely been performed clinically for decades (Flake and Zanjani, 1999; Troeger et al., 2006). Indeed, to date, 46 *in utero* transplants have been performed in human patients (Tiblad and Westgren, 2008; Tarantal and Lee, 2010), for 14 different genetic disorders, including 1 case of HA (Troeger et al., 2006; Touraine, 2013). These studies have collectively provided unassailable proof that the early human fetus can be accessed multiple times with an extremely low procedure-related risk, assuming that a minimally invasive, ultrasound guided approach is employed (Flake et al., 1996; Flake and Zanjani, 1999; Tarantal et al., 2006; Troeger et al., 2006; Merianos et al., 2008; Roybal et al., 2010; Tarantal and Lee, 2010). It is important to note that it was studies performed in the fetal sheep model that provided the experience and knowledge that led to the first curative *in utero* transplant in a human patient (Flake et al., 1996), emphasizing the value and importance of the fetal sheep model for developing clinically viable approaches to therapy, and for predicting clinical outcome. Using these established, clinically proven methods to deliver a corrective FVIII gene early in gestation could cure HA *in utero*, enabling the birth of a normal healthy baby requiring no further treatments. Such a treatment, if successful, would clearly represent a major advance, both from an economic standpoint (one treatment rather than a lifetime of expensive treatments several times each week), and with respect to the quality of life of the patient.

While most individuals with a family history of HA are encouraged to have prenatal screening (~70–75% of new HA cases arise in families with a history of HA), parents presented with a prenatal diagnosis of HA currently have only 2 possible choices: pregnancy termination or the birth of a child with HA. The availability of a safe and effective *in utero* treatment would provide parents a much-needed 3rd option, which would certainly provide the needed impetus for much more widespread prenatal HA screening. In contrast to *in vitro* embryo screening and selection, which has been proposed as a possible solution in families with a history of HA and other genetic diseases, *in utero* gene therapy requires only minimal equipment that would already be in place for prenatal diagnosis, and should not be prohibitively expensive. Several recent studies have provided conclusive evidence that prenatal screening for the hemophilias can be cost-effective, even when considering developing third world countries (Klein et al., 2001; Sasanakul et al., 2003; Peyvandi, 2005). Moreover, another recent study has shown it is now possible to diagnose HA *in utero* by performing digital PCR on the small number of fetal cells present within the mother's peripheral blood, making it possible to diagnose HA prenatally with essentially zero risk to the fetus or mother (Tsui et al., 2011).

Although the clinical and financial advantages of *in utero* gene therapy are compelling, in and of themselves, it is important to realize that there are also features of the fetus that make it a better gene therapy recipient than the adult (Matzinger, 2002; Porada et al., 2004a,b). For instance, cell populations that are quiescent in the adult, and largely refractory to transduction with many commonly employed viral vectors, are actively cycling in the fetus and amenable to transduction at relatively high efficiencies. For example, we showed that a single intraperitoneal injection of a small volume of γ-retroviral vector resulted in gene transfer levels within the hematopoietic system of 5–6% (Porada et al., 1998, 2001a, 2002a; Tran et al., 2000); levels that would undoubtedly be beneficial in HA. Further studies involving antibody selection of CD34^+^ cells and serial transplantation/repopulation (Porada et al., 1998, 2008; Tran et al., 2000), provided compelling evidence that this approach successfully modified bona fide hematopoietic stem cells, indicating this method could provide lifelong disease correction.

Our results also demonstrated that this approach successfully transduced hepatocytes and hepatic endothelium at levels that could well be therapeutic in HA, and defined the temporal window during gestation for optimal transduction of these cells within the liver (Porada et al., 2005a). Concurrently, fetal gene delivery experiments conducted in sheep, rodent, and non-human primate models, by other investigators who employed a variety of viral-based vectors, produced similar results (Porada et al., 1998, 2002b, 2004a, 2005a,b; Lipshutz et al., 1999, 2000; Schneider et al., 1999, 2002; Themis et al., 1999; Tarantal et al., 2001a,b,c, 2005, 2006; David et al., 2003; Waddington et al., 2003, 2004; Chen et al., 2004a; Jimenez et al., 2005; Lee et al., 2005; Park et al., 2009; Tarantal and Lee, 2010). The collective results of these studies clearly support the ability of this method to deliver a FVIII transgene to the nascent liver with sufficient efficiency to convert severe HA patients to a moderate or, perhaps, even mild phenotype (Porada et al., 2005a).

While the active cell cycling in the fetus enables efficient transduction with vectors that require mitosis, it is important to note that this ongoing proliferation in all of the fetal organs is also of benefit when using vectors that do not have an absolute requirement for mitosis. Gene delivery early in gestation, regardless of the vector employed, also makes it possible to achieve subsequent expansion of these gene-corrected cells throughout the rest of gestation. As such, even if the initial gene transfer only transduces a small number of the desired target cells, this subsequent expansion could produce clinically useful levels of gene-correction by birth.

As mentioned earlier, one of the biggest obstacles/drawbacks to treating severe HA by repeated infusion of purified or recombinant FVIII protein is the formation of inhibitory antibodies in ~30% of patients. It is important to note that there are also distinct immunologic benefits to performing gene therapy in the developing fetus. Early in immunologic development, before thymic processing of mature lymphocytes, the fetus appears to be highly receptive to foreign antigens. Indeed, exposure to foreign antigens during this period often results in sustained tolerance, which can become permanent if the presence of the antigen is maintained (Billingham et al., 1954). We have spent the last two decades performing *in utero* gene transfer studies in the sheep model (Porada et al., 1998, 2001a,b, 2004a, 2005a; Tran et al., 2000; Park et al., 2003a,b, 2004), and have shown that it is possible to take advantage of this unique temporal window of immuno-naïveté to deliver exogenous genes during this period of gestation and induce durable tolerance to the vector-encoded gene product (Tran et al., 2001). This tolerance induction appears to involve both cellular and humoral mechanisms, since antibody and cellular responses to the transgene product were both significantly diminished in these animals, even several years after fetal gene transfer. Indeed, further mechanistic studies demonstrated that gene delivery early in fetal development exploits several central and peripheral tolerogenic avenues that exist in the fetus (Colletti et al., 2008). These results strongly imply that fetal gene therapy, even if it does not cure HA, would still be an ideal treatment modality for this disease, since permanent immune tolerance to FVIII could be induced. This would thus ensure that postnatal therapy, be it protein- or gene-based, could proceed safely without any of the immune-related problems that currently plague HA treatment.

To-date, the only experimental studies to directly investigate fetal gene therapy for the treatment of the hemophilias have targeted hemophilia B (factor IX deficiency) (Lipshutz et al., 1999; Schneider et al., 1999, 2002; Themis et al., 1999; David et al., 2003, 2011; Waddington et al., 2003, 2004; Chen et al., 2004a; Mattar et al., 2011). This is most likely a result of the greater ease with which FIX can be cloned into a variety of viral vectors, and efficiently expressed upon transduction of appropriate target cells; this is in marked contrast to the difficulties that were initially seen when attempting to express FVIII in the context of viral vectors (Ponder, 2011). Because HA patients have at least a ten-fold higher likelihood of developing inhibitors than hemophilia B patients (Ehrenforth et al., 1992; Chitlur et al., 2009), these studies, while encouraging, leave unanswered the critical question of whether fetal gene delivery's ability to induce immune tolerance to marker gene products and FIX will hold true for the induction of tolerance to FVIII, given FVIII's higher inherent immunogenicity. We are currently addressing is important question in the sheep model.

All of the afore-referenced studies demonstrated that the direct injection of viral vectors into the developing fetus can be an effective way of delivering an exogenous gene and achieving long-term expression in multiple tissues and confirmed the therapeutic potential of an *in utero* approach to gene therapy. However, for this direct vector injection method of fetal gene delivery to move forward into the clinical arena, vectors that can target specific cell types will likely need to be developed, to eliminate the risk of off-target modification of undesirable non-target cells, like those of the germline (Park et al., 2004, 2009; Lee et al., 2005). Since such vectors are currently not available, we have been testing the ability of mesenchymal stromal cells (MSC) to serve as vehicles for delivering genes to the developing fetus to safely correct HA and other diseases prior to birth. In the next section of this chapter, we will discuss our rationale for using these cells as therapeutics and summarize results to-date following *in utero* delivery of MSC.

## Mesenchymal stromal cells (MSC) as HA therapeutics

Decades after the pioneering studies of Friedenstein on the marrow microenvironment (Friedenstein et al., 1974; Friedenstein, 1991), results of studies from various labs around the world have revealed that mesenchymal stromal cells (MSC) possess a very broad differentiation potential, both *in vitro* and *in vivo*, and exhibit properties that suggest that at least some of the cells contained within this population may be stem cells (Caplan, 1991; Liechty et al., 2000; Mackenzie and Flake, 2001; Fukuda, 2002; Jiang et al., 2002; Airey et al., 2004; Chen et al., 2004b; Kassem, 2004; Porada et al., 2006; Banas et al., 2007; Chamberlain et al., 2007; Colletti et al., 2009a; Porada and Almeida-Porada, 2010). MSC are very rare, only comprising roughly 0.001–0.01% of cells within the marrow (Galotto et al., 1999). However, they can be passaged extensively *in vitro* without a loss of differentiative potential, making it possible to readily generate clinically relevant numbers of these cells (Crop et al., 2009). Since MSC were first discovered within the bone marrow, many of the studies performed thus far have utilized MSC isolated from this tissue. However, we and others have now shown that cells with the phenotype and functionality of MSC can also readily be isolated from a variety of different tissues, including umbilical cord blood, kidney, liver, lung, brain, fetal blood, and even adipose tissue collected via liposuction (Zuk et al., 2001, 2002; Almeida-Porada et al., 2002; Morizono et al., 2003; in 't Anker et al., 2003; Lee et al., 2004a; Fan et al., 2005; Gotherstrom et al., 2005). Importantly from the standpoint of *in utero* therapies, MSC have also been isolated from the amniotic fluid and the chorionic villi, raising the exciting possibility that autologous MSC could be used as cellular therapeutics or gene delivery vehicles for *in utero* therapy (Poloni et al., 2011; Shaw et al., 2011a,b; Fernandes et al., 2012; Karlsson et al., 2012; Weber et al., 2012).

As discussed earlier, the liver is thought to be the body's main source of FVIII. Studies from our group and others over the past decade have provided compelling evidence that MSC from various sources can give rise, *in vitro* and *in vivo*, to cells which appear identical to native hepatocytes, and have shown that transplanting MSC in a range of model systems results in the generation of substantial numbers of hepatocytes, with resultant repair/correction in a variety of inborn genetic defects and injuries (Almeida-Porada et al., 2001a, 2003a, 2004; Schwartz et al., 2002; Theise and Krause, 2002; Almeida-Porada and Zanjani, 2004; Fang et al., 2004; Sakaida et al., 2004; Lee et al., 2004b; Luk et al., 2005; Sato et al., 2005; Zhao et al., 2005; Ishikawa et al., 2006; Oyagi et al., 2006; Popp et al., 2006; Talens-Visconti et al., 2006; Aurich et al., 2007, 2008; Banas et al., 2007, 2008, 2009; Chamberlain et al., 2007; Colletti et al., 2007, 2009b; Higashiyama et al., 2007; Muraca et al., 2007; Sgodda et al., 2007; di Bonzo et al., 2008; Enns and Millan, 2008; Lysy et al., 2008; Zheng and Liang, 2008). We have shown, in the fetal sheep model, that, by performing the transplant at a stage in early gestation when the fetal immune system is still relatively immature, it is possible to achieve significant levels of human cell engraftment. Moreover, because this approach induces donor-specific tolerance, these xenogeneic human cells persist for the whole life of the transplanted animals (Almeida-Porada et al., 2001a, 2004; Almeida-Porada and Zanjani, 2004). Of direct relevance to HA treatment, we have demonstrated that, after transplantation into fetal sheep, human MSC engraft at levels of up to 12% within the recipient liver (Almeida-Porada et al., 2000, 2001b, 2003b, 2004; Almeida-Porada and Zanjani, 2004; Chamberlain et al., 2004, 2007; Porada and Almeida-Porada, 2006, 2010), and contribute to both the parenchyma and the perivascular zones, placing them in an ideal location to deliver FVIII into the circulation. Since FVIII levels of only 3–5% of normal would convert a patient with severe HA to a moderate or mild phenotype, it seems reasonable to conclude that these levels of engraftment should be highly therapeutic. In other recent studies, we have demonstrated that MSC from various tissues throughout the body endogenously produce and secrete biologically active FVIII (Soland et al., 2014). Collectively, these results support the notion that MSC are uniquely and ideally suited for treating HA.

However, upon further analysis, we found that, although MSC engrafted at significant levels within the natural sites of FVIII synthesis, the levels of FVIII production were too low to effectively treat HA. If, however, one were to use gene transfer to engineer MSC to express FVIII, it is likely that the levels of MSC engraftment we routinely achieve following transplantation *in utero* would be beneficial/therapeutic in HA, especially if newer, expression-optimized FVIII variants were used in the gene therapy vectors (Gangadharan et al., 2006; Doering et al., 2009; Dooriss et al., 2009; Ide et al., 2010). Importantly, MSC can be efficiently transduced with all of the major viral vector systems that are in clinical use, including adenovirus (Bosch et al., 2006; Bosch and Stice, 2007; Roelants et al., 2008), murine retroviruses (Meyerrose et al., 2007; Sales et al., 2007; Piccoli et al., 2008; Roelants et al., 2008; Gnecchi and Melo, 2009), lentiviruses (Zhang et al., 2002, 2004; Meyerrose et al., 2008; Fan et al., 2009; Wang et al., 2009; Xiang et al., 2009), and AAV (Kumar et al., 2004; Stender et al., 2007). Furthermore, in contrast to studies with hematopoietic stem cells (Racine et al., 1995; Fox and Chowdhury, 2004; Muraca and Burlina, 2005), human MSC are stable in culture, do not form tumors *in vivo* (unlike murine MSC, Tasso et al., 2009), and there is no evidence that transduction can cause human MSC to undergo transformation or progression to clonal dominance. Instead, recent studies have shown that, even following intentional induction of genomic instability, human MSC undergo terminal differentiation rather than transformation (Altanerova et al., 2009), with very rare transformants only arising after very extended *in vitro* propagation, and being easily identifiable (and removable) based on their altered cell surface marker profile (Pan et al., 2014). As such, MSC appear to represent very safe cellular vehicles for delivering a therapeutic gene.

Looking specifically at using MSC to treat HA, multiple studies have already proven that MSC can be efficiently transduced with murine retroviral and lentiviral vectors with gene cassettes encoding FVIII from a variety of species and produce/release high levels of functional FVIII protein. Importantly, when FVIII was purified from the tissue culture medium of transduced MSC, its specific activity, electrophoretic mobility, and proteolytic activation pattern were all identical to commercially produced FVIII (Doering, 2008). Given the widespread distribution and engraftment of MSC following their transplantation, the ability of MSC to give rise, *in vivo*, to cells of numerous tissue types, and their ability to efficiently process and secrete significant quantities of biologically active FVIII, it is not surprising that we and others feel that MSC represent ideal vehicles for delivering a FVIII transgene throughout the body, and thereby providing long-term/permanent correction of HA (Van Damme et al., 2003; Doering, 2008; Pipe et al., 2008; Porada et al., 2011).

In addition to their widespread engraftment and their ability to serve as delivery vehicles for the FVIII gene, MSC have rather unique immunological properties that may further increase their utility for treating HA. MSC do not normally express MHC class II or the co-stimulatory molecules CD80 and CD82. As a result, they do not induce allogeneic lymphocytes to proliferate, nor do they serve as very effective targets for cytotoxic T cells or NK cells. Actually, a growing body of evidence exists to support the conclusion that MSC can be transplanted across allogeneic barriers without eliciting a pronounced immune response (Bartholomew et al., 2001; Devine et al., 2001). Thus, it is theoretically possible that HA (and other diseases as well) could be treated using “off-the-shelf” MSC from an unrelated donor, which would greatly facilitate the use of these cells for therapy. It has long been presumed that the immune system is immature/absent in the fetus at the time when *in utero* transplant is performed. However, recent studies conducted in mice by Mackenzie and Flake have challenged this assumption by showing that the engraftment rate of allogeneic hematopoietic cells can be negatively affected by not only the developing fetal immune system, but also by that of the mother (Peranteau et al., 2007; Nijagal et al., 2011). As such, the success of *in utero* therapies may also benefit from the hypoimmunogenic state of MSC.

In addition to their unique immune properties, MSC also possess another interesting characteristic that is potentially of great clinical value; the ability to selectively migrate to sites within the body where injury/inflammation exist. Upon arriving at these sites, the MSC then repair the damaged/diseased tissue by: (1) releasing trophic factors that dampen inflammation and stimulate the local tissue's endogenous repair mechanisms; and (2) engrafting within the target tissue and reprogramming to produce tissue-specific cells (Jiang et al., 2005, 2006a,b). This property raises the exciting possibility that, following infusion, FVIII-expressing MSC might have the ability to selectively traffic to active bleeds/sites of injury, thus directing the therapy to regions of the body that are in greatest need of help.

## Preclinical success with MSC-based treatment for HA

Despite the multiple advantages of early intervention, there are already roughly 1.6 × 10^∧^4 individuals with HA, in the US alone, who obviously could not benefit from the development of a therapy that would be administered prior to birth. Furthermore, over 25% of the mutations that cause HA arise *de novo*; as such, it is highly improbable that this group of patients would undergo prenatal screening for HA. We therefore began investigating, in two pediatric HA lambs, whether some of the afore-mentioned properties that make MSC ideally suited for delivering FVIII could still be realized if the MSC engineered to express FVIII are delivered early in childhood. During their first months of life, both HA lambs in this study were given frequent human FVIII treatments in an effort to control multiple hematomas and recurring bleeds in the leg joints, that had given rise to chronic, progressive, debilitating hemarthroses. As a result of these joint bleeds, the two sheep developed extreme postural and gait defects, which made it difficult for them to even stand upright, and precluded them from walking. Given the severe, life-threatening phenotype of the HA sheep, we chose to use cells from the “father” of the two HA lambs (haploidentical), rather than attempting to collect marrow from the two HA lambs to isolate autologous MSC.

Based on our prior *in utero* studies, we knew that MSC should engraft throughout all of the major organs (Feldmann et al., 1992; Almeida-Porada et al., 2004; Aurich et al., 2006; Russo et al., 2006; Chamberlain et al., 2007; Colletti et al., 2009a) and durably express the vector-encoded genes (Feldmann et al., 1992; Zanjani et al., 1993; Colletti et al., 2009a) following intraperitoneal (IP) injection. The IP route also enabled the MSC to gradually enter the circulation over an extended period of time, as they were absorbed through the peritoneal lymphatics, rather than as a single large bolus, as would occur via IV injection. The IP route also allowed us to avoid the extensive lung-trapping which occurs following IV administration of MSC, promising more efficient delivery of the MSC to the desired target tissues, and eliminating the clinical risk of emboli formation (Traas et al., 2007; Mancuso et al., 2009).

MSC were simultaneously transduced with 2 lentiviral-based vectors; one of which contained a cassette coding for an expression/secretion optimized porcine FVIII (pFVIII) transgene (Yamagami et al., 2006), and the second of which encoded eGFP, to enable us to follow/trace the donor cells *in vivo* following injection. Two factors drove our choice to use a pFVIII transgene: (1) the cDNA for ovine FVIII had not yet been cloned; and (2) prior studies had shown that, even when expressed in human cells, the pFVIII transgene was produced/secreted at levels that were 10–100 times higher than those seen with hFVIII (Gangadharan et al., 2006; Doering et al., 2007, 2009). As such, we anticipated that even if the transplanted MSC only engrafted/persisted at relatively low levels, they should still be able to exert a clinical benefit. Once the transduced MSC had been sufficiently expanded, transduced MSC were transplanted into the peritoneal cavity of the first animal under ultrasound guidance, without any prior preconditioning of the recipient.

At various time points after transplantation, a highly sensitive chromogenic assay was unable to detect any FVIII activity in the circulation of this animal, but his clinical picture was markedly improved within only days of the transplant. The animal stopped experiencing spontaneous bleeds, and he enjoyed an event-free clinical course, no longer requiring hFVIII infusions. What was most remarkable, however, was that the animal's joints recovered fully. His existing hemarthroses resolved, restoring normal posture and gait, and allowing him to resume a normal activity level. This is the first report describing phenotypic correction of severe HA in a large animal model after transplanting cells modified to express FVIII. It is also the first time that chronic debilitating hemarthroses have been reversed as a result of any type of therapeutic intervention.

Encouraged by this first animal's marked improvement, we performed an identical transplant procedure on a second animal, using a four-fold higher cell dose, in the hopes of achieving detectable FVIII activity in the circulation. Just as had occurred in the first animal, this straightforward procedure resolved existing hemarthroses in this second animal, and he promptly regained normal activity. The transplant also enabled this second animal to achieve factor-independence. These results thus confirm the ability of MSC to serve as highly effective cellular vehicles for delivery a FVIII transgene, and establish their ability to exert a pronounced clinical benefit in this large animal HA model. Nonetheless, the plasma of this second animal, just like that of the first animal, was completely devoid of FVIII activity. As such, the question of the mechanism(s) whereby this procedure produced such clear therapeutic benefit remains unanswered.

Following euthanasia, PCR analysis of tissues collected from these animals confirmed widespread engraftment of significant levels of MSC in all of the tissues we analyzed, including liver, lymph nodes, intestine, lung, kidney, omentum, and thymus. Subsequent analysis of frozen tissue sections by confocal microscopy revealed large numbers of MSC had engrafted within the synovia of the joints that were experiencing hemarthrosis at the time of transplant. Moreover, these MSC were still expressing the vector-encoded FVIII transgene. These analyses thus confirmed the intrinsic ability of transplanted MSC to home to and persist within sites of ongoing injury/inflammation. Furthermore, the continued production/release of FVIII by the engrafted cells, locally within the joint, provided a mechanistic explanation for the pronounced improvement this procedure exerted on the animals' joints. However, the long-term presence of FVIII-expressing MSC within the animals' joints cannot really account for the clear systemic benefits we observed in these animals, the most striking of which was their complete cessation of spontaneous bleeding events.

Confocal microscopy also demonstrated that transplanted MSC had engrafted within the small intestine, in agreement with what we had seen in our prior *in utero* studies (Feldmann et al., 1992). Since proteins secreted from cells within the intestine should have fairly easy access to the circulation, future studies designed to enhance the levels of intestinal engraftment could likely produce a dramatic improvement in the amount of FVIII that is released into the systemic circulation. Aside from the intestine and injured/diseased joints, MSC engraftment also occurred in the liver, the lungs, and the thymus of the treated animals. Collectively, the results of the PCR and confocal analyses strongly support the conclusion that widespread durable engraftment of MSC can be achieved in a large animal model following transplantation in a postnatal setting, without the need for preconditioning/ablation. However, the levels of engraftment seen in these pediatric animals were substantially lower than those obtained in our prior *in utero* studies. Moreover, despite the marked clinical improvement and the widespread engraftment of the transplanted MSC, both animals mounted a strong immune response to pFVIII, which agrees with prior studies conducted in HA mice (Gangadharan et al., 2006). These inhibitors exhibited cross-reactivity to hFVIII, which was unanticipated, since a good deal of clinical data exist to support the continued efficacy of pFVIII products in human patients that have developed anti-hFVIII inhibitors (VandenDriessche et al., 1999; Brown and Lillicrap, 2002; Bhakta et al., 2006; Son et al., 2006).

Therefore, while this postnatal approach proved that MSC can serve as cellular vehicles to deliver a FVIII transgene and produce a therapeutic benefit, we feel it is safe to conclude that administering this same treatment *in utero* would have a more pronounced and more durable effect, since higher levels of donor MSC engraftment could be achieved, and because inhibitor formation could be avoided due to the induction of immune tolerance to the FVIII transgene.

### Conflict of interest statement

The authors declare that the research was conducted in the absence of any commercial or financial relationships that could be construed as a potential conflict of interest.
